# Hypertrophic Cardiomyopathy and Phenocopies: New Therapies for Old Diseases—Current Evidence and Future Perspectives

**DOI:** 10.3390/jcm14124228

**Published:** 2025-06-13

**Authors:** Maria Alfarano, Federico Ciccarelli, Giulia Marchionni, Federico Ballatore, Jacopo Costantino, Antonio Lattanzio, Giulia Pecci, Silvia Stavagna, Leonardo Iannelli, Gioacchino Galardo, Carlo Lavalle, Fabio Miraldi, Carmine Dario Vizza, Cristina Chimenti

**Affiliations:** Department of Clinical, Internal, Anaesthesiology and Cardiovascular Sciences, Sapienza University of Rome, 00161 Rome, Italy; m.alfarano@policlinicoumberto1.it (M.A.); federico.ciccarelli@uniroma1.it (F.C.); giulia.marchionni.1@studenti.unipd.it (G.M.); federico.ballatore@uniroma1.it (F.B.); jacopo.costantino@uniroma1.it (J.C.); antonio.lattanzio@uniroma1.it (A.L.); pecci.1747716@studenti.uniroma1.it (G.P.); silvia.stavagna@uniroma1.it (S.S.); leonardo.iannelli@uniroma1.it (L.I.); gioacchino.galardo@uniroma1.it (G.G.); carlo.lavalle@uniroma1.it (C.L.); fabio.miraldi@uniroma1.it (F.M.); dario.vizza@uniroma1.it (C.D.V.)

**Keywords:** cardiomyopathy, genetic test, cardiac magnetic resonance, disease-modifying therapy

## Abstract

The hypertrophic cardiomyopathy (HCM) clinical phenotype includes sarcomeric HCM, which is the most common form of inherited cardiomyopathy with a population prevalence of 1:500, and phenocopies such as cardiac amyloidosis and Anderson–Fabry disease, which are considered rare diseases. Identification of cardiac and non-cardiac red flags in the context of multi-organ syndrome, multimodality imaging, including echocardiography, cardiac magnetic resonance, and genetic testing, has a central role in the diagnostic pathway. Identifying the specific disease underlying the hypertrophic phenotype is very important since many disease-modifying therapies are currently available, and phase 3 trials for new treatments have been completed or are ongoing. In particular, many chemotherapy agents (alkylating agents, proteasome inhibitors, immunomodulatory drugs, and monoclonal antibodies targeting clonal cells) allowing one to treat AL amyloidosis, transthyretin stabilizers (tafamidis and acoramidis), and gene silencers (patisiran and vutrisiran) are available in transthyretin cardiac amyloidosis, and enzyme replacement therapies (agalsidase-alpha, agalsidase-beta, and pegunigalsidase-alpha) or oral chaperone therapy (migalastat) can be used in Anderson–Fabry disease. In addition, the introduction of cardiac myosin inhibitors (mavacamten and aficamten) has deeply modified the treatment of hypertrophic obstructive cardiomyopathy. The aim of this review is to describe the new disease-modifying treatments available in HCM and phenocopies in light of current scientific evidence.

## 1. Introduction

The hypertrophic cardiomyopathy (HCM) clinical phenotype is a structural and functional heart muscle disorder characterized by left ventricular (LV) wall thickness ≥ 15 mm in any myocardial segment that is not explained by loading conditions and recognizes different etiologies, such as sarcomeric HCM; it is the most common inherited cardiomyopathy and includes phenocopies such as Anderson–Fabry disease (AFD) and cardiac amyloidosis (CA), which represent 5–10% of all HCM diagnoses [1,2].

Multimodality imaging, including echocardiography, cardiac magnetic resonance (CMR), and genetic testing, has a central role in the diagnostic pathway. Moreover, CMR allows for tissue characterization through late gadolinium enhancement (LGE) imaging and mapping techniques. The presence of LGE in the most hypertrophied segment, increased extracellular volume (ECV) and T1 mapping values are associated with prognosis [3].

The 2023 ESC Guidelines for the Management of Cardiomyopathies highlight the importance of a broad phenotype-based approach including morphological characterization with multimodality imaging, genetic testing, and identification of cardiac and non-cardiac red flags that can contribute to recognizing HCM phenocopies as an expression of systemic diseases such as short PR interval, cornea verticillate, and angiokeratomas in AFD or low peripheral voltages on ECG, macroglossia, and carpal tunnel syndrome in CA.

It is essential to promptly recognize the HCM phenotype because there are specific therapies based on etiology. Disease-modifying therapies are currently available in CA, such as chemotherapy agents (alkylating agents, proteasome inhibitors, immunomodulatory drugs, and monoclonal antibodies targeting clonal cells), in AL amyloidosis, transthyretin stabilizers (tafamidis and acoramidis), and gene silencers (patisiran and vutrisiran), in transthyretin CA and in AFD, such as enzyme replacement therapy (ERT) (agalsidase-alpha, agalsidase-beta, and pegunigalsidase-alpha) and oral chaperone therapy (migalastat). The introduction of cardiac myosin inhibitors (CMIs) has deeply modified the treatment of obstructive hypertrophic cardiomyopathy (oHCM).

The aim of this review is to describe the new disease-modifying treatments available in HCM and phenocopies in light of current scientific evidence (graphical abstract).

A literature review was conducted using MEDLINE and PubMed databases. The search terms included the following: cardiomyopathies, HCM, CA, AFD, LV hypertrophy, and disease-modifying therapy.

## 2. Hypertrophic Cardiomyopathy

HCM was first described in 1958 by Teare, a pathologist in London, as hamartomas [4]. Initially considered a rare disease, nowadays, HCM is the most common inherited cardiomyopathy with a worldwide prevalence of 1:500 [5], and it is characterized by an LV hypertrophy unexplained by loading conditions. In 40% of cases, HCM is due to a mutation of sarcomeric genes, and it is transmitted as an autosomal dominant trait with incomplete penetrance and variable expressivity [6]. The sarcomeric genes strongly associated with HCM are those encoding myosin heavy chain beta (*MYH7*, OMIM: 160760) and myosin-binding protein C 3 (*MYBPC3*, OMIM: 600958), followed by cardiac troponin I 3 (*TNNI3*, OMIM: 191044), cardiac troponin T 2 (*TNNT2*, OMIM:191045), tropomyosin alpha-1 chain (*TPM1*, OMIM: 191010), myosin light chain 2 (*MYL2*, OMIM: 160781), myosin light chain 3 (*MYL3*, OMIM: 160790), and actin alpha cardiac muscle 1 (*ACTC1*, OMIM: 102540) [2]. These genes represent a large percentage of the genetic causes of HCM, but there are also other less common variants that contribute to the genetic diversity of this disease. Cardiomyopathies, including HCM, are often linked to mutations in genes that encode sarcomere proteins, which are the fundamental structures of cardiac muscle fibers. Gerull highlights how this genetic complexity makes genetic analysis important for accurate diagnosis and personalized patient management [7]. Understanding the genetic “landscape” also helps identify at-risk family members and plan more effective monitoring and treatment strategies.

Histologically, features of HCM are myocyte hypertrophy and disarray, interstitial and replacement fibrosis, wall thickening, and dysfunction of intramyocardial vessels [8]. The clinical presentation is extremely variable. Some patients are asymptomatic or mildly symptomatic. The most common symptoms are dyspnea on exertion, palpitation, fatigue, pre-syncope, and angina [9]. Significant complications are atrial fibrillation, syncope, heart failure, ventricular arrhythmias, and sudden death [10]. About 2/3 of HCM patients have left ventricular outflow tract (LVOT) obstruction (LVOTO) that contributes to morbidity [11]. The better understanding of the HCM pathophysiology has led to the development of CMIs (mavacamten and aficamten), a novel drug class that has been shown in recent trials to reduce LVOTO, symptoms, and NYHA class and improve exercise tolerance in oHCM.

### 2.1. Current and Emerging Therapies in oHCM

The obstructive form of HCM is identified by a gradient in LVOT > 30 mmHg [1]. However, the hemodynamic impact of LVOTO occurs with a peak gradient > 50 mmHg [1,2].

Current and emerging therapies in oHCM and non-oHCM are synthetized in Table 1.

Standard treatment of oHCM is based on non-vasodilating beta-blockers [12] and non-dihydropyridine calcium channel blockers [13]. Through their negative chronotropic and inotropic effects, beta-blockers and calcium channel blockers prolong the diastole, determine myocardium relaxation, improve LV filling, and reduce LVOTO. If symptoms persist despite therapy with beta-blockers or calcium channel blockers titrated to the maximum dosage, disopyramide is added as a second-line therapy [14]. Disopyramide is a class IA antiarrhythmic, i.e., a sodium channel antagonist. The effect on LVOTO is due to the negative inotropic effect of the drug. Although beta-blockers, calcium channel blockers, and disopyramide are the standard therapy for oHCM, their efficacy has not been demonstrated in large randomized clinical trials. Moreover, these drugs do not act on molecular mechanism of LVOTO. Furthermore, attention should be paid to side effects, such as sinus arrest and atrioventricular block with beta-blocker and calcium channel antagonists, postural hypotension with calcium channel antagonists due to the effect on the smooth muscle of the vessels resulting in vasodilation, which can also worsen LVOTO, and anticholinergic side effects and QT prolongation with disopyramide.

CMIs are allosteric inhibitors of cardiac-specific myosin, a novel and effective therapeutic option for adult patients with symptomatic NYHA Classes II and III oHCM [2,15]. Available drugs include mavacamten (approved for the treatment of symptomatic obstructive HCM in 2022 by the FDA and in 2023 by the EMA) and aficamten (pending approval for the same indication). CMIs act on the pathophysiology of oHCM by blocking the excess hyperactivation of cardiac myosin. In normal conditions, there is a balance between the “on” and “off” state of cardiac myosin heads; in oHCM, there is dysregulation between the “on” and “off” state in favor of the “on” due to overactivation of the enzyme adenosine triphosphatase (ATPase), resulting in excessive activation of the sarcomere, impaired relaxation, and waste of energy. CMIs reduce energy expenditure, actomyosin crossbridge formation, and hypercontractility, as demonstrated in preclinical studies [16,17]. Mavacamten stabilizes myosin in a low-energy-consuming conformation. Aficamten has a similar mechanism of action, although it has a longer half-life and fewer interactions with cytochrome P450. Phase III randomized clinical trials (RCT) have shown that CMIs are effective in oHCM, resulting in a reduction in LVOTO, circulating cardiac markers NTproBNP and high-sensitive (hs) troponin, and improvement in quality of life and exercise capacity, as well as diastolic function. CMIs can lead to a reduction in LV ejection fraction (LVEF). If LVEF is <50%, the drug should be discontinued for 4 weeks. The reduction in LVEF is usually transient and reversible with temporary suspension of the treatment and does not require hospitalization.

The PIONEER-HCM study [18] was an open-label, non-randomized, phase 2 trial to assess the safety and efficacy of mavacamten in 21 symptomatic oHCM patients. Patients in cohort A received 10 or 15 mg/day of mavacamten, with dose titration to 20 mg/day at week 4 based on LVEF evaluation. In cohort B, patients received 2.5 mg/day of mavacamten with dose titration to 5 mg/day in cases of LVOT gradient reduction < 50% from baseline after 4 weeks. The trial demonstrated that mavacamten can significantly reduce LVOTO (*p* = 0.008) and improve exercise capacity and symptoms in patients with oHCM; moreover, the molecule can reduce LVEF in a concentration-dependent manner, as plasma concentrations above 695 ng/mL are associated with LVEF reduction between 34% and 49%. In the PIONEER-OLE (PIONEER Open-Label Extension) study [19], the safety and effectiveness of mavacamten for >3 years was evaluated in oHCM patients who had previously completed the PIONEER-HCM study.

Based on the encouraging results of PIONEER-HCM, EXPLORER-HCM was designed. EXPLORER-HCM [20] is a pivotal phase 3, double-blind, placebo-controlled, randomized trial that evaluated the efficacy of mavacamten in 251 symptomatic oHCM patients in NYHA class II or III compared to placebo, in addition to medical therapy with beta-blockers or calcium antagonists; treatment with disopyramide was not permitted because of its additive negative inotropic effect in association with mavacamten. The primary endpoint was a composite of exercise capacity and symptom burden at week 30 compared with baseline, defined as a ≥1.5 mL/kg/min increase in pVO2 and ≥1 NYHA class reduction or a ≥3.0 mL/kg/min improvement in pVO2 and no worsening of the NYHA class. In case of LVEF reduction < 50%, the drug was temporarily discontinued. The primary endpoint was achieved in 37% of patients in the mavacamten group versus 17% in the placebo group (*p* = 0.0005). Regarding secondary endpoints, patients in the mavacamten group showed a greater reduction in the post-exercise LVOT gradient (*p* < 0.0001), greater increase in pVO2 (*p* = 0.0006), and improved symptom scores evaluated with the Kansas City Questionnaire Overall Summary Score (KCCQ-OSS) (*p* < 0.0001) than patients in the placebo group. Also, NTproBNP and hs-Troponin were reduced in the mavacamten group. Long-term efficacy and safety of mavacamten was confirmed in the LTE study of EXPLORER-HCM (EXPLORER-LTE) [21], part of the 5-year MAVA-LTE (A Long-Term Safety Extension Study of Mavacamten in Adults Who Have Completed MAVERICK-HCM or EXPLORER-HCM) program [22]. Among the 244 patients who had completed EXPLORER-HCM, 231 (95%) were enrolled in EXPLORER-LTE. An interim analysis showed that clinical improvement in the LVOT gradient and NYHA functional class and reduction in serum cardiac biomarkers (NTproBNP and hs-Troponin) remained stable after a median 62-week follow-up. Twelve patients (5.2%) developed transient left ventricular dysfunction (LVEF < 50%) that was resolved with temporary discontinuation of mavacamten.

VALOR-HCM [23] is a randomized, double-blind, placebo-controlled, phase 3 trial that evaluated the efficacy of mavacamten versus placebo in 112 patients eligible for SRT. After 16 weeks of treatment, 76.8% of patients in the placebo group versus 17.9% of patients in the mavacamten group were still eligible for septal reduction therapy (SRT) (*p* < 0.001). Therefore, mavacamten reduced the need for SRT, and this effect, as well as improvement in LVOT gradients and symptoms, were confirmed after 56 weeks of follow up [24].

Aficamten showed similar effects on LVOTO, NYHA class, symptoms, and reduction in serum cardiac biomarkers in the phase 2 REDWOOD-HCM trial [25]. In the recent SEQUOIA study [26], a phase 3, double-blind trial, 282 patients were randomized to aficamten or placebo for 24 weeks. The primary end point was the change from baseline to week 24 in the peak oxygen uptake as assessed by a cardiopulmonary exercise test. At week 24, the average increase in pVO2 was 1.8 mL/kg/min (95% confidence interval [CI], from 1.2 to 2.3) in the aficamten group versus 0.0 mL/kg/min (95% CI, from −0.5 to 0.5) in the placebo group (*p* < 0.001). All secondary end points (tested hierarchically—in particular, KCCQ-CSS changes, improvement in the NYHA functional class, change in the pressure gradient after the Valsalva maneuver, occurrence of a gradient of less than 30 mm Hg after the Valsalva maneuver, and the duration of eligibility for SRT—all assessed at week 24) significantly improved in the aficamten group compared to the placebo group.

**Figure 1:**
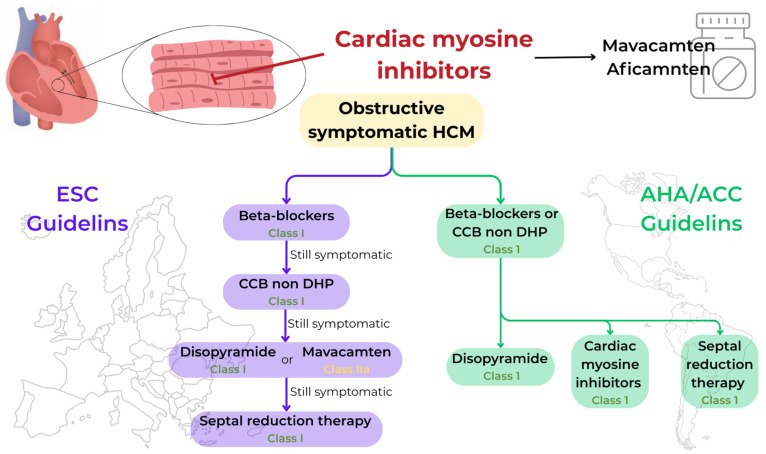
Flowchart of the treatment of obstructive HCM: differences between ESC guidelines and AHA/ACC guidelines regarding the indication for cardiac myosin inhibitors.

Based on these results, the current ESC guidelines [2] place mavacamten in class IIa for the treatment of symptomatic oHCM, while the recent AHA/ACC guidelines [15] place CMIs in class I for the same indication (Figure 1).

In patients who remain symptomatic or are refractory to medical therapy, SRT, either septal myectomy or alcohol septal ablation [27,28], is recommended. ESC guidelines [2] suggest mavacamten as an intermediate option compared to surgery; instead, AHA/ACC guidelines [14] consider CMIs on the same level, as an alternative to invasive therapies.

There are two ongoing phase 3 studies evaluating the potential benefit of mavacamten and aficamten in symptomatic patients with non-obstructive HCM (ODISSEY-HCM [29] and ACACIA-HCM, NCT06081894, respectively) and two smaller studies on pediatric patients with symptomatic obstructive HCM (SCOUT-HCM [NCT06253221] with mavacamten, and CEDAR-HCM [NCT06412666] with aficamten).

RTCs on other drugs effective against HCM including losartan [30], ranolazine [31], spironolactone [32], and sacubitril/valsartan [33] have not demonstrated any efficacy in improving functional capacity. Otherwise, sodium-glucose co-transporter-2 (SGLT2) inhibitors in patients with HCM are associated with improved survival and cardiovascular symptoms and reduced hospitalizations [34].

### 2.2. Practice Implications for oHCM

- HCM is the most common inherited cardiomyopathy with a worldwide prevalence of 1:500.
- Clinical presentation includes heart failure, atrial fibrillation, syncope, ventricular arrhythmias, and sudden death.
- Standard treatment of oHCM is based on non-vasodilating beta-blockers and non-dihydropyridine calcium channel blockers. If symptoms persist despite therapy with beta-blockers or calcium channel blockers titrated to the maximum dosage, disopyramide is added as second-line therapy.
- CMIs (mavacamten and aficamten) are a novel drug class that has been shown in recent trials to reduce LVOTO, symptoms and NYHA class and improve exercise tolerance in oHCM.

### 2.3. Cardiac Amyloidosis

CA is a restrictive cardiomyopathy caused by the extracellular deposition of amyloid fibrils formed by misfolded proteins into the heart. The first case of CA was described by Sokya in 1876 [35]. In 1965, Pomerance estimated the prevalence of so-called ‘senile’ cardiac amyloidosis of 10% in subjects aged >80 years and 50% in subjects aged >90 years [36]. Initially considered a rare and fatal disease, CA is a widely recognized disease nowadays. The advancement of knowledge in terms of pathophysiology, the improvement of diagnostic techniques, and the development of ‘disease-modifying’ therapies have changed the natural course of CA. Although 30 amyloidogenic proteins are known, in 95% of cases CA is caused by the deposition of misfolded monoclonal immunoglobulin light chain (AL) derived from an abnormal clone of plasma cell proliferation (AL amyloidosis [37]) or misfolded transthyretin (TTR), a protein synthesized by the liver and involved in the transport of the hormone thyroxine and retinol-binding protein (TTR amyloidosis [38]). TTR amyloidosis (ATTR) can be hereditary in cases of misfolded mutated TTR (ATTR-v) or non-hereditary in cases of misfolded wild-type (wt) TTR (ATTR-wt). Amyloidosis is a systemic disease, but cardiac involvement drives morbidity and mortality. Life expectancy is 2.5–3.5 years if the disease is left untreated, but early diagnosis, better management, and specific therapy have profoundly changed the natural history of the disease [39].

AL amyloidosis has a prevalence of 1–2 cases per 100,000 subjects [40]. In a recent metanalysis, ATTR-wt cardiomyopathy (ATTRwt-CM) was found to be present in 12% of patients with HF with preserved ejection fraction (HFpEF), 10–15% of elderly patients with severe aortic stenosis undergoing valve replacement, and 7% of patient with a hypertrophic phenotype of cardiomyopathy. Therefore, ATTR-wt seems to be anything but a rare disease and it is more common in the elderly and in males; on the contrary, ATTR-v is certainly a rare disease characterized by adult onset with an estimated global prevalence of 5000–10,000 persons [41]. ATTR manifests mainly with cardiomyopathy and neuropathy [42]. Deposition of TTR amyloid fibrils in the heart leads to heart failure (usually HFpEF), restrictive cardiomyopathy, and arrhythmias. The accumulation of TTR amyloid in the nervous system results in peripheral sensorimotor polyneuropathy and autonomic dysfunction. ATTR deposition in soft tissue causes tunnel carpal syndrome, trigger finger, spontaneous rupture of the biceps brachii tendon, and cervical and lumbar spinal stenosis [41].

**Figure 2:**
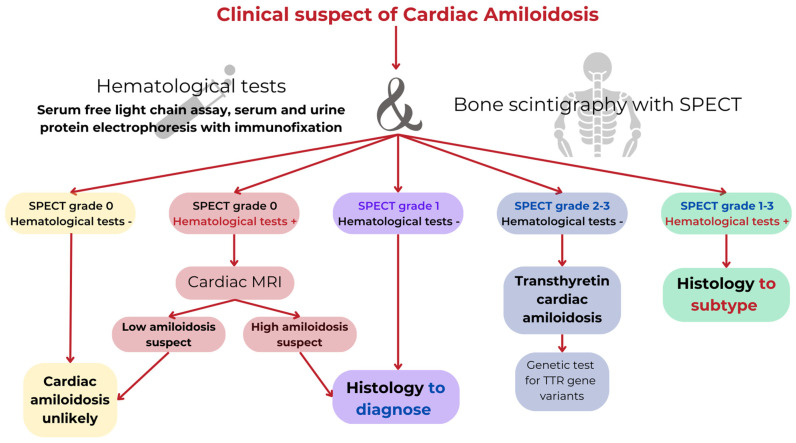
Diagnostic flowchart in CA. In the presence of signs and symptoms, electrocardiographic, echocardiographic, or CMR features suggestive of CA, the diagnostic pathway includes non-invasive criteria, i.e., bone scintigraphy combined with planar and single-photon emission computed tomography (SPECT) and exclusion of a clonal dyscrasia through serum free light-chain assays, serum, and urine protein electrophoresis with immunofixation. In cases of positive bone scintigraphy (Perugini grade 2 or 3) and the exclusion of monoclonality, a TTR genetic test is required to distinguish ATTRv from ATTRwt; otherwise, if the Perugini grade is 1 or there is a positive immunofixation, histological confirmation (cardiac or extracardiac) is mandatory.

CA should be suspected in patients > 65 years of age with LV wall thickness ≥ 12 mm and the presence of cardiac and extracardiac red flags (such as autonomic dysfunction, peripheral polyneuropathy, skin bruising, bilateral carpal tunnel syndrome, reduced longitudinal strain with apical sparing on 2D echocardiography, decreased QRS voltage to mass ratio, and/or pseudo Q waves on ECG) [43]. In the presence of signs and symptoms, electrocardiographic, echocardiographic, or CMR features suggestive of CA, the diagnostic pathway includes non-invasive criteria, i.e., bone scintigraphy combined with planar and single-photon emission computed tomography (SPECT) and exclusion of a clonal dyscrasia through serum free light-chain assays, serum, and urine protein electrophoresis with immunofixation (Figure 2). In cases of positive bone scintigraphy (Perugini grade 2 or 3) and exclusion of monoclonality, a TTR genetic test is required to distinguish ATTRv from ATTRwt; otherwise, if the Perugini grade is 1 or there is positive immunofixation, histological confirmation (cardiac or extracardiac) is mandatory [2,42] (Figure 2).

### 2.4. Current and Emerging Therapies in CA

The understanding of pathophysiological mechanisms has led to the development of disease-modifying therapies that have made CA, initially considered fatal, a treatable disease nowadays.

Current and emerging therapies in AL amyloidosis and ATTR are reported in Table 2.

In AL amyloidosis, current guidelines recommend a combination of cyclophosphamide, bortezomib, and dexamethasone (CyBorD) and daratumumab as first-line therapy; autologous stem cell transplantation (ASCT) is considered in eligible patients (about 20% of cases), especially those who do not achieve a satisfactory response to combination therapy [44]. Hematological efficacy is very high with a very good partial response in 78% of patients; however, about 20% of patients discontinue at least one treatment, and about 30% of patients reduce therapy due to side effects [45].

In ATTR-CM, there are three types of therapies that block the amyloidogenic cascade at different points: TTR stabilizers (tafamidis, diflunisal and acoramidis), TTR suppressors through gene silencing (patisiran, vutrisiran, eplontersen) and gene editing (NTLA-2001 based on clustered, regularly interspaced, short palindromic repeats [CRISPR]-Cas9 technology), and degraders of amyloid fibrils (monoclonal antibodies such as NI006/ALX2220, coramitug, and AT02) [46]. At the moment, tafamidis has been approved in several countries; acoramidis has been recently approved by the Food and Drug Administration (FDA) and the European Medicines Agency (EMA); vutrisiran has been recently approved by the FDA and EMA; and eplontersen and degraders are under investigation. The 2021 ESC HF guidelines place tafamidis in the class I recommendation group for the treatment of ATTRv-CM and ATTRwt-CM patients in NYHA classes I or II to reduce symptoms, cardiovascular hospitalizations, and mortality [47].

### 2.5. Stabilizers of TTR

Tafamidis is a TTR stabilizer that binds to the T4 binding site, and its efficacy was evaluated in ATTR-ACT, the first trial for the treatment of ATTR-CM. Tafamidis was associated with a 30% relative reduction in mortality with a number needed to treat (NNT) of 7.5 to prevent one death. Moreover, tafamidis reduced cardiovascular and all-cause hospitalizations from 0.7 per year to 0.48 per year, with a NNT of 4 to prevent one cardiovascular hospitalization over a year [48]. Kaplan–Meier survival curves began to diverge after approximately 18 months of treatment. Tafamidis was also associated with a significant lower rate of decline in distance during the 6 min walking test (6MWT) (*p* < 0.001) and in KCCQ (*p* < 0.001). An interim analysis of the LTE of ATTR-ACT demonstrated that patients initially treated with tafamidis in ATTR-ACT had substantially better survival rates than those first treated with placebo, highlighting the importance of early diagnosis and treatment in ATTR-CM [49]. An additional post hoc analysis of the LTE study demonstrated that in patients aged <80 years, tafamidis was associated with longer median survival (80 vs. 41 months; HR = 0.4513, [95% CI: 0.3176–0.6413]; *p* < 0.0001) than those initially treated with placebo [50]. Patients aged ≥80 years treated continuously with tafamidis showed a longer median survival rates, but these were not statistically significant (45 vs. 27 months; all-cause mortality HR = 0.6828 [95% CI: 0.4048–1.1517]; *p* = 0.1526) in comparison to those initially treated with placebo. In a recent international, multicenter, cohort study, tafamidis was associated with lower mortality after propensity score matching on variables including age, National Amyloidosis Centre (NAC) stage, and NYHA class, also in octogenarians (*p* = 0.053) [51]. Tafamidis treatment seems to be futile in patients aged ≥90 years and with NAC stage ≥ 3.

Diflunisal is a nonsteroidal anti-inflammatory drug that stabilizes TTR by binding to the T4 binding site. Data on diflunisal efficacy in ATTR-CM come from single-center studies with limited numbers of patients [52]. It can be an alternative in countries that cannot afford the costs of other medications. Due to its anti-inflammatory effect, diflunisal may worsen hypertension and cause gastrointestinal bleeding and renal failure. Monitoring of renal function is recommended in patients taking diflunisal.

Acoramidis is a highly selective stabilizer of TTR and a more potent stabilizer compared to tafamidis [53].

In the ATTRibute-CM trial, 632 ATTR-CM patients were randomized (2:1) to acoramidis (800 mg twice a day) or placebo for 30 months [54]. The primary four-step hierarchical analysis (including all-cause mortality, cumulative frequency of cardiovascular hospitalizations, change in NTproBNP and in 6MWT at 30 months) was in favor of acoramidis (*p* < 0.001); side effects were similar in the two groups. In the OLE of the ATTRibute-CM trial [55], patients who previously received acoramidis continued the drug, and patients who initially received placebo were switched to acoramidis. The trial confirmed the clinical benefit of acoramidis compared to placebo in terms of all-cause mortality and cardiovascular hospitalization at 42 months of follow up (HR = 0.57 [95% CI, 0.46–0.72], *p* < 0.0001).

### 2.6. TTR Suppressors: Gene Silencers

Patisiran is a first-generation small interfering RNA (siRNA) approved for ATTRv with polyneuropathy in 2018 following the APOLLO-A trial, which demonstrated its efficacy on polyneuropathy after 18 months of treatment. Patisiran is administered intravenously every 21 days and requires premedication to reduce infusion reactions. In the APOLLO A trial, some patients also had cardiomyopathy, and the administration of patisiran was associated with an improvement in global longitudinal strain (GLS) and a reduction in LV wall thickness and NTproBNP [56]. Therefore, in APOLLO B, a phase 3, double-blind, randomized trial, 360 ATTRv-CM and ATTRwt-CM patients were randomized to patisiran or placebo in a 1:1 ratio for 12 months [57]. Patisiran was associated with a lower decline in 6MWT (*p* = 0.02) and KCCQ (*p* = 0.04). However, no significant benefits were observed for the second secondary end point (a composite of death from any cause, cardiovascular events, and change from baseline in the 6MWT over 12 months).

Vutrisiran is a second-generation TTR siRNA conjugated to N-acetyl galactosamine (GalNAc). Compared to patisiran, vutrisiran has the advantage of subcutaneous administration every 4 months and does not require premedication. In the HELIOS-B trial, 655 ATTR-CM patients in NYHA class I-III, 40% of whom were taking tafamidis, were randomized to vutrisiran or placebo [58]. Vutrisiran showed a lower risk of death from any cause and recurrent cardiovascular events in comparison to placebo (HR in the overall population, 0.72; 95% CI 0.56 to 0.93; *p* = 0.01; HR in the monotherapy population, 0.67; 95% CI, 0.49 to 0.93; *p* = 0.02) and a lower risk of death from any cause through 42 months. Both in the overall population and monotherapy population, vutrisiran was associated with a lower decline in 6WMT and KCCQ.

Eplontersen is a novel GalNAc-conjugated antisense oligonucleotide (ASO) with the same basic sequence as inotersen, which does not cause glomerulonephritis and thrombocytopenia. It is administered subcutaneously every 4 weeks. The CARDIO-TTRansform trial (NCT04136171) evaluating the efficacy and safety of eplontersen in ATTR-CM patients is ongoing.

### 2.7. TTR Suppressors: (CRISPR)-Cas9 Technology

Gene editing therapy using CRISPR-Cas9 technology has been applied to ATTR. Nexiguran ziclumeran (Nex-Z), formerly NTLA-2001, is a vivo gene-editing therapeutic agent targeting the gene encoding TTR. A phase I open-label trial evaluated the safety and efficacy of a single dose of NTLA-2001 in six patients with ATTRv with polyneuropathy, at the dose of 0.1 mg per kilogram and 0.3 mg per kilogram [59]. After 28 days, the group that received the dose of 0.1 mg per kilogram showed a reduction in TTR serum concentration of 52%, while the group that received the dose of 0.3 mg per kilogram showed a reduction in TTR serum concentration of 87%. MAGNITUDE (NCT06128629) is a phase III clinical trial currently recruiting ATTR-CM patients that will be randomized to NTLA-2001 or placebo in a 2:1 ratio; concomitant treatment with a stabilizer is possible, but, on the contrary, gene silencers are not allowed.

### 2.8. Degraders of Amyloid Fibrils

Trials of monoclonal antibodies capable of removing amyloid fibrils, such as ALX2220 (formerly NI006) [60], coramitug (formerly NNC6019–0001) [61], and AT-02 [62], are ongoing.

### 2.9. Practice Implications for CA

- CA is a restrictive cardiomyopathy caused by extracellular deposition of amyloid fibrils formed by misfolded proteins into the heart.
- In 95% of cases, CA is caused by the deposition of misfolded monoclonal immunoglobulin light chains derived from an abnormal clone of plasma cell proliferation (AL amyloidosis) or misfolded transthyretin (TTR amyloidosis).
- The improvement of diagnostic techniques and the development of ‘disease-modifying’ therapies have changed the natural course of CA.
- Chemotherapy agents (alkylating agents, proteasome inhibitors, immunomodulatory drugs, and monoclonal antibodies targeting clonal cells) are available in AL amyloidosis.
- In ATTR-CM, there are three types of therapies that block the amyloidogenic cascade at different points: TTR stabilizers (tafamidis, diflunisal, and acoramidis), TTR suppressors through gene silencing (patisiran, vutrisiran, eplontersen) and gene editing (NTLA-2001 based on CRISPR-Cas9 technology), and degraders of amyloid fibrils (monoclonal antibodies such as NI006/ALX2220, coramitug, and AT02).

## 3. Anderson–Fabry Disease

**Figure 3:**
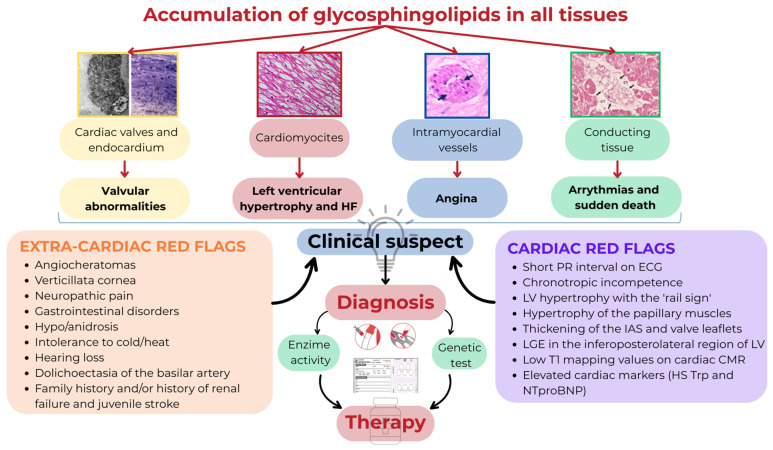
Diagnostic pathway in AFD.

AFD is a rare X-linked lysosomal disorder caused by mutations in the GLA gene (Xq21.3-q22) encoding for the α-galactosidase A (α-Gal A), a lysosomal hydrolase involved in the catabolism of glycosphingolipids [63]. The deficient or absent activity of α-Gal A leads to the accumulation of glycosphingolipids, primarily globotriaosylceramide (Gb3) and its derivative globotriaosylsphingosine (lyso-Gb3), in the vascular endothelium of a wide variety of tissues, including the kidney, heart, central and peripheral nervous system, skin, and gastrointestinal tract [64]. AFD was first described simultaneously by dermatologist Johannes Fabry [65] and the surgeon William Anderson [66] in 1898. The estimated prevalence of the classic form of AFD is 1 in 40,000 to 60,000 adult males [63]. However, neonatal screening programs have reported a high prevalence of pathogenic variants ranging from 1 in 1250 to 1 in 7800 [67,68]. AFD can be distinguished in a *classic* form and a *non-classic*, late-onset form [69]. The classic phenotype is characterized by severely reduced or absent activity of the enzyme α-Gal A and manifests from early childhood with neuropathic pain, verticillata cornea, gastrointestinal symptoms, hypohidrosis, hearing loss, and angiokeratomas. The late-onset phenotype is less severe and shows residual activity of the enzyme α-Gal A, usually manifesting in the third-to-fourth decade of life and primarily affecting the kidneys and/or the heart. In the heart, the accumulation of glycosphingolipids involves all cell types—cardiomyocytes, endocardium, intramyocardial vessels, valves, and conduction tissue—leading to the development of hypertrophic cardiomyopathy, heart failure, angina, valvular abnormalities, bradyarrhythmias and tachyarrhythmias, and sudden death. AFD accounts for 0.5–1% of HCM cases, and its correct recognition can be a challenge, as both cardiac and extracardiac manifestations of the disease need to be identified (Figure 3). Cardiac red flags include a short PR interval on ECG, chronotropic incompetence, LV hypertrophy with the ‘rail sign’, hypertrophy of the papillary muscles, thickening of the interatrial septum and valve leaflets on 2D echocardiography, LGE in the inferoposterolateral region of LV, low T1 mapping values on cardiac CMR, and elevated cardiac markers (HS Troponin and NTproBNP). The extracardiac red flags are angiocheratomas, verticillata cornea, neuropathic pain, gastrointestinal disorders, hypo/anidrosis, intolerance to cold/heat, hearing loss, dolichoectasia of the basilar artery, a family history and/or history of renal failure, and juvenile stroke.

In cases of diagnostic suspicion, the diagnosis is simple and is based on α-Gal A enzyme activity measurement in blood leukocytes or dried blood spot testing [70] (Figure 3). In males with the classic phenotype, an undetectable α-Gal A activity or activity below 3% of the expected value is diagnostic. Although the measurement of α-Gal A activity is diagnostic in males, genetic testing is essential to confirm the disease and for genotype–phenotype correlation. In females, residual enzymatic activity may be normal due to X chromosome inactivation; therefore, genetic testing is necessary for diagnosis. When a proband is identified, family genetic screening should be initiated. Clinical monitoring is crucial to assess disease progression and requires a multidisciplinary approach. In a recent study, a novel staging system for classifying cardiac damage in patients with AFD has been proposed. Based on echocardiographic parameters, the authors identified four stages correlated with cardiovascular outcomes: stage 0 if no cardiac involvement; stage 1 with LV hypertrophy (LV maximal wall thickness > 12 mm); stage 2 with left atrium (LA) enlargement (LA volume index > 34 mL/m^2^); and stage 3 with ventricular impairment defined by LV ejection fraction < 50% or E/e′ ≥ 15 or tricuspid annular plane systolic excursion < 17 mm [71]. In another study, FD cardiomyopathy (FDCM) was classified in four stages (stage 0: non-hypertrophic; stage 1: hypertrophic–pre-fibrotic; stage 2: hypertrophic–fibrotic; and stage 4: overt dysfunction) according to clinical manifestation and laboratory, electrocardiographic, and echocardiographic features [72]. Future validation studies for the staging of FDCM are necessary in order to achieve correct prognostic stratification.

### 3.1. Current and Emerging Therapies in AFD

The main goal of AFD treatment is to prevent disease progression. Optimal management of AFD patients requires a multidisciplinary approach. Approved specific treatments include ERT and chaperone therapy (migalastat), while new therapeutic approaches are under development (Table 3). ERT was approved in Europe by the EMA starting in 2001, with two different pharmaceutical formulations for intravenous administration: agalsidase-alpha, produced from human fibroblasts, and agalsidase-beta, derived from Chinese hamster ovary cells. In the USA, the FDA has only approved the agalsidase-beta formulation. Both enzymatic formulations contain recombinant human α-Gal but are produced in different cell lines and use different genetic engineering techniques. This results in different glycosylation and sialylation profiles of the glycoproteins, so their therapeutic efficacy in approval studies was tested at different dosages: 0.2 mg/kg every other week for agalsidase-alpha and 1 mg/kg every other week for agalsidase-beta [73,74]. ERT has profoundly changed the natural history of AFD and improved patients’ quality of life through the effective treatment of neuropathic pain, gastrointestinal manifestations, heat intolerance, and exercise intolerance [75]. Long-term follow-up studies and registry data show that ERT delays the progression of heart disease and reduces the rate of cardiovascular events [76,77]. ERT is well tolerated and stabilizes intracellular Gb3 deposits; however, complete removal is difficult even after long-term therapy. Several factors influence the cardiac response, including phenotype, sex, age at treatment initiation, and the development of anti-drug antibodies (ADAs) against the exogenous α-Gal A enzyme. Additionally, the presence of an immune-mediated inflammatory process can limit its effectiveness [78]. In cases of advanced disease with severe LVH and myocardial fibrosis, the effect of ERT is limited [79].

Migalastat is a chaperone molecule approved by the EMA (2016) and by the FDA (2018) for AFD patients aged 12 years and older with estimated glomerular filtration rates > 30 mL/min/1.73 m^2^ and with ‘amenable’ GLA variants, at the dose of 123 mg orally every other day. Migalastat is an imino-sugar that, by binding to the catalytic site of α-GAL A, stabilizes the enzyme and promotes its transport inside the lysosomes. The amenable mutations are characterized by the presence of residual α-Gal A activity of at least 3% of normal and an increase in activity of at least 20% in the presence of 20 μM migalastat in cultured patient lymphocytes. Among the 1000 pathogenic variants associated with AFD, approximately 30–50% (mainly missense mutations) are considered amenable to therapy with migalastat. The efficacy and safety of migalastat have been demonstrated in two RCTs: ATTRACT [80] and FACETS [81]. Recent real-world data have shown a significant discrepancy between the susceptibility predicted in vitro and the actual increase in vivo of α -Gal A enzyme activity and clinical response in certain genetic variants [82]. This may be related to intrinsic limitations of the in vitro susceptibility test and the possible dose-dependent inhibitory effects of migalastat. These findings suggest that the biochemical and clinical response to chaperone therapy should be carefully monitored to confirm clinical efficacy.

Migalastat is safe and generally well tolerated; the most common side effects are headache, diarrhea, procedure pain, and vertigo.

Both ATTRACT and FACET trials demonstrated a significant decrease in LV mass index (LVMi) in patients treated with migalastat [81,82]. This has also been confirmed by the real-world FAMOUS trial that assessed the efficacy and safety of migalastat in 60 patients previously treated with ERT or ERT-naïve. Mean LVMi decreased significantly by 10.2 (95% CI 5.3–15.2) g/m^2^ (108.6 ± 48.0 vs. 98.4 ± 41.4 g/m^2^, *p* = 0.001) both in males and females [83]. However, cardiac data were evaluated through 2D transthoracic echocardiography with well-known limitations of both inter- and intra-observer variability. Recently, the MAIORA trial evaluated the effectiveness of migalastat on cardiac involvement in 16 treatment-naïve patients (4 women) with FDCM using CMR [83]. It did not demonstrate a significant reduction in myocardial mass after 18 months but rather its stabilization, with an increase in T1 mapping at the septal level, indicating a possible disease stabilization at the myocardial level.

A novel PEGylated recombinant alpha-galactosidase produced in plant cells, pegunigalsidase alfa, was approved in 2023 by the FDA and EMA for intravenous administration at the dose of 1 mg/kg every other week in AFD adult patients. Produced in a ProCellEx^®^ system, alpha-galactosidase has increased stability, prolonged half-life, and reduced immunogenicity due to pegylation [84].

In the BRIDGE study, 22 patients, previously treated with agalsidase alfa, received pegunigalsidae alpha for 12 months showing an improvement in the mean annualized eGFR slope in both male and female patients (from −5.90 mL/min/1.73 m^2^/year on agalsidase alpha to −1.19 mL/min/1.73 m^2^/year on pegunigalsidase alfa).

In the BRIGHT study, 29 patients previously treated with agalsidase alfa or beta every other week for ≥3 years were switched to 2 mg/kg pegunigalsidase alfa every 4 weeks for 52 weeks. No patients developed de novo ADAs during treatment. The median eGFR slope was −1.9 mL/min/1.73 m^2^/year from baseline.

The FDA and EMA approved pegunigalsidase alpha in accordance with the results of BALANCE, a randomized, double-blind, head-to-head phase 3 noninferiority study comparing pegunigalsidase alfa and agalsidase beta in AFD patients previously treated with agalsidase beta for more than 1 year with worsening of renal function [85]. The difference in the median slope of eGFR between pegunigalsidase alpha and agalsidase beta was −0.36 mL/min/1.73 m^2^/year (95%CI −2.44, 1.73), meeting the prespecified non-inferiority margin. ADAs were detected in 15% of pegunigalsidase alfa-treated patients and 26% of agalsidase beta-treated patients. Minimal changes in LysoGB3 concentrations were observed in both groups. Adverse events and mild or moderate infusion-related reactions were similar in both groups.

Substrate reduction therapy (SRT) is based on the oral administration of imino-sugars that directly inhibit the synthesis of glycosphingolipids. These drugs, previously validated in Gaucher disease, can be administered regardless of the AFD genotype. Several oral formulations of SRT are currently being tested in clinical studies [86,87].

Preclinical, in vivo gene therapy studies in α-Gal A knockout mouse models using adenoviral and lentiviral vectors have demonstrated a dramatic increase in α-Gal A activity and a significant reduction in lysoGb3 [88,89].

Messenger RNA (mRNA) therapies are experimental and currently being tested on primates and rats. The target is hepatocytes, where the α-Gal A enzyme is produced and secreted from the inoculated mRNA delivered via lipid vectors administered intravenously. The enzyme produced in this way is endogenous, with glycosylation profiles that do not trigger any autoimmune response [90].

### 3.2. Practice Implications for AFD

- AFD is a rare X-linked lysosomal disorder caused by mutations in the GLA gene.
- The main goal of AFD treatment is to prevent disease progression.
- Specific approved treatments include ERT (agalsidase-alpha, agalsidase-beta, and the recently approved pegunigalsidase alfa) and chaperone therapy (migalastat).
- New therapeutic approaches including substrate reduction therapy, gene therapy, and mRNA therapies are under development.

## 4. Conclusions and Future Directions

The 2023 ESC Guidelines for the Management of Cardiomyopathies highlighted the importance of a broad phenotype-based approach. Identifying the underlying disease is mandatory, since disease-modifying therapies are currently available in oHCM, CA, and AFD. Future studies are needed to understand the effectiveness of CMIs in the early stages of the oHCM, in non-obstructive HCM, and in HFpEF.

The current availability of various disease-modifying therapies for ATTR-CM will make comparative studies necessary for combination therapy, determining the timing of starting treatment in carriers, and evaluating cost-effectiveness in fragile patients with advanced stages of the disease.

Finally, new therapeutic options, as well as risk stratification systems, are currently being studied for ATTR-CM, as well as for AFD.

## Figures and Tables

**Table 1:** Current and emerging therapies in obstructive and non-obstructive HCM. * obstructive hypertrophic cardiomyopathy; ** dihydropyridine; # left ventricular outflow obstruction; ## atrial fibrillation; § left ventricular ejection fraction; §§ atrio-ventricular.

Therapy (Category)	oHCM *	Non-oHCM	Notes
Non-vasodilating Beta-Blockers	First-line therapy for symptom relief and gradient reduction	Used for angina or arrhythmias	
Calcium Channel Blockers (non-DHP **)	Commonly used as alternative to beta-blockers. Avoid in case of severe LVOTO #	Used for diastolic dysfunction and rate control	Use verapamil or diltiazem; avoid dihydropyridines in oHCM *
Disopyramide	Used as add-on therapy for drug-refractory obstructive symptoms	Not used.	Positive effect as rhythm control strategy in AF ##; anticholinergic effects limit use. QT interval monitoring is required.
Myosin Inhibitor—Mavacamten and Aficamten	Second-line for symptomatic oHCM *	Not approved	Requires LVEF § monitoring. Excellent response and safety as stand-alone therapy
Ranolazine	Not routinely used—off-label in selected cases.	Reduce symptoms linked to microvascular angina	RESTYLE-HCM trial suggests possible benefit on diastolic function. Antiarrhythmic effect is beneficial
Valsartan	Use with caution	Investigational—potential disease-modifying role	VANISH trial: slowed progression in early-stage sarcomeric HCM
Septal Myectomy	First line for drug-refractory LVOTO # or LVOTO # obstruction primarily caused by mitral valve alterations	Not applicable	Excellent outcomes in high-volume centers
Alcohol Septal Ablation	Yes—alternative to surgery for selected patients	Not applicable	Minimally invasive; risk of AV §§ block
Heart Transplantation	For end-stage, drug-refractory cases	Considered—for restrictive/dilated progression	Early candidacy is key

**Table 2:** Current and emerging therapies in cardiac amyloidosis. * transthyretin amyloidosis; ** transthyretin; # small interfering RNA; ## hereditary transthyretin amyloidosis with polyneuropathy; § transthyretin cardiac amyloidosis; §§ cardiovascular; // antisense oligonucleotides; *** clustered regularly interspaced short palindromic repeats and CRISPR-associated protein 9; ^ light chain amyloidosis; ### cyclophosphamide, bortezomib, and dexamethasone; §§§ autologous stem cell transplant.

Amyloid Type	Therapy	Mechanism/Class	Status and Key Clinical Evidence
A-TTR *	Tafamidis	TTR ** stabilizer	Approved for hereditary and wild-type forms. ATTR-ACT trial: reduced mortality and hospitalization.
	Acoramidis	TTR ** stabilizer	Approved 2024. ATTRibute-CM trial showed functional and biomarker benefit.
	Patisiran	siRNA # TTR ** silencer	Approved for hATTR-PN ##; APOLLO-B showed preserved functional capacity.
	Vutrisiran	siRNA # TTR ** silencer	Approved 2024 for ATTR-CM §. HELIOS-B showed reduced CV §§ events.
	Inotersen	ASO // TTR ** silencer	Approved for hATTR-PN ##.
	Eplontersen	ASO // TTR ** silencer	Approved for hATTR-PN #. CARDIO-TTRansform trial for isolated cardiac involvement is ongoing.
	NTLA-2001	CRISPR-Cas9 *** gene editing	Investigational. Phase I showed 90% TTR ** knockdown.
AL ^	Daratumumab + CyBorD ###	Anti-CD38 + chemotherapy	Approved. ANDROMEDA trial showed deep hematologic and organ responses.
	ASCT §§§ + Melphalan	High-dose chemo + stem cell	Standard in eligible patients. Durable remissions.
	CAEL-101	Anti-amyloid monoclonal antibody	Investigational. Aims to clear cardiac AL ^ deposits.

**Table 3:** Current and emerging therapies in Anderson–Fabry Disease. * globotriaosylceramide; ** European Union; *** left ventricular.

Therapy	Mechanism/Class	Target	Status and Key Clinical Evidence
Agalsidase beta (Fabrazyme^®^)	Enzyme replacement	Systemic	Approved. Reduces Gb3 *; long-term studies show stabilization of renal and cardiac function.
Agalsidase alfa (Replagal^®^)	Enzyme replacement	Systemic	Approved in EU **/Canada. Stabilizes LV *** mass and kidney function.
Pegunigalsidase alfa (Elfabrio^®^)	Enzyme replacement (PEGylated)	Systemic	Approved in 2023. Longer half-life and reduced immunogenicity.
Migalastat (Galafold^®^)	Pharmacologic chaperone	Systemic	Approved for amenable mutations. Oral route.
Venglustat	Substrate reduction therapy	Systemic	Investigational. Reduces plasma Gb3 *; ongoing trials.
Lucerastat	Substrate reduction therapy	Systemic	Investigational. Reduced Gb3 *; failed pain endpoint.
ST-920	Gene therapy (liver-directed)	Systemic	Investigational. Sustained enzyme activity post-single dose.
4D-310	Gene therapy (cardiac-directed)	Cardiac	Investigational. Early studies show effective cardiac gene uptake.
